# Toxic Effect of Khat in Rat Embryos and Fetuses

**DOI:** 10.1155/2021/9933389

**Published:** 2021-07-29

**Authors:** Selamawit Belete, Kaleab Asres, Yonas Bekuretsion, Rekik Ashebir, Melese Shenkut Abebe, Girma Seyoum

**Affiliations:** ^1^Department of Anatomy, College of Health Sciences, Addis Ababa University, Addis Ababa, Ethiopia; ^2^Department Pharmaceutical Chemistry and Pharmacognosy, College of Health Sciences, Addis Ababa University, Addis Ababa, Ethiopia; ^3^Department of Pathology, College of Health Sciences, Addis Ababa University, Addis Ababa, Ethiopia; ^4^Traditional and Modern Medicine Research Directorate, Ethiopian Public Health Institute, Addis Ababa, Ethiopia

## Abstract

Khat (*Catha edulis* Forsk) is a plant consumed by many people in Eastern Africa, including Ethiopia, and Southern Arabia to be stimulated. There are several human and animal studies on khat that provide information about its toxic effects. However, the potential toxic effects of khat on embryos and fetuses have not been elucidated. The aim of the present study was to investigate the embryotoxic and fetotoxic effects of khat exposure during the earliest period of gestation in rats. Pregnant Wistar albino rats were treated with khat extract at 250, 500, and 750 mg/kg doses from day 6 through day 12 of gestation. The treatment was delivered by gavage. Embryos and fetuses were recovered on gestational day 12 or day 20, respectively, and were quantitatively and qualitatively assessed for developmental anomalies. Placentae from the treatment and control groups were investigated for histopathological effects. Results of the present study showed that khat exposure during pregnancy had dose-dependent toxic effects in rat embryos and fetuses. Prenatal growth retardation such as reduced fetal weight and crown-rump length was observed in near-term fetuses, especially, in animals treated with the highest dose of khat (*p* < 0.05). Growth retardation and developmental anomalies were also observed in day 12 embryos of khat-treated rats. Maternal weight gain of the khat-treated group was also significantly lower than the control group. Cytolysis, decidual hypoplasia, and atrophy were observed in the placenta of the khat-treated rats. Findings of the present study revealed, for the first time, that exposure of pregnant rat to crude extract of khat causes embryotoxic and fetotoxic effects.

## 1. Introduction

The consumption of khat (*Catha edulis* Forsk) is extensive in Ethiopia. It is also widely consumed in eastern Africa, including the Horn of Africa [1]. In Ethiopia, the productive age group of the population is the most consumer of khat. Khat is easily found in different parts of Ethiopia. Recent studies indicate that there was a significant increase in the number of Ethiopians that chew khat [2]. In the earlier time, khat has been grown primarily in the eastern part of Ethiopia; however, currently, it is commonly cultivated in most parts of the country. Khat consumption, traditionally restricted to a certain group of the population, had become accepted among all segments of the population. Khat chewing often leads to the use of illegal substances [2].

Khat has been consumed for as long as thousands of years for different purposes. The fresh leaves of khat contain a central nervous system stimulant (cathinone). By this, it is mainly used as a recreational drug which helps to attain a state of euphoria and stimulation [3]. Khat users usually become talkative, alert, elated, aggressive, hyperactive, and euphoric. On the other hand, chewing khat, mainly in North-Eastern Africa and the Arabian Peninsula, is a social and cultural tradition [4].

Based on recent research reports, consuming khat is now a worldwide phenomenon; users are now found in Europe and North America as well [5]. Khat uses adversely affects the nervous system. Significant association was reported between khat consumption and the presence of anxiety and depression [4]. Moreover, schizophreniform psychotic illness, mania, increased alertness, dependence, and other psychiatric symptoms were noticed in individuals who consumed khat [3]. Khat users also encounter anorexia, constipation, malaise, and gastritis [6–9]. Other toxic effects such as sleeplessness, headache, increased blood pressure and heart rate, restlessness, and impaired sexual potency in men also were observed in khat users [8–10]. A research conducted in Haromaya district, Eastern Ethiopia, reported that there was a significant association between frequent khat use and developing anemia. Moreover, the presence of tannins in khat leaves reduces the physiological activity of nonheme iron from the food [11].

Earlier study report indicates that the prevalence of khat chewing was higher in male compared with female, whereas, recent reports, in some countries, revealed sharp escalation in khat use by females. In Ethiopia, recent estimates of khat chewing prevalence reach up to 50%, with 17% self-described as daily users, predominantly men (a reported 5 : 1 male to female ratio) [12]. Recent researches conducted in East Africa indicate that 80–90% of males and 10–60% of females are chewing khat daily. This data indicates that gender difference in khat consumption is becoming narrow [13].

Based on a study conducted in Yemen, on 1141 pregnant women, the prevalence of low-birth-weight babies was higher in khat chewer mothers compared with nonchewers [14]. There is one report of a pregnant woman developing tachycardia, hypertension, and chest pain after a khat chewing session [15]. However, there are no other published data of any adverse effects related to khat chewing during pregnancy.

Although there are data on different adverse effects of khat, very little is known about its effects on the embryos/fetuses. Therefore, the main purpose of this study is to evaluate the embryotoxicity and fetotoxicity of khat in rats.

## 2. Materials and Methods

### 2.1. Experimental Design

The experiments are designed to study the embryotoxicity/fetotoxicity of khat in Wistar rats. Experiments were carried out in day 20 fetuses (day 20 experiment) and in day 12 embryos (day 12 experiment). We followed the methods of Seyoum and Persaud [16], Seyoum [17], and Teshome et al. [18]. Animals were treated with different doses of khat for a period of one week, from day 6 through day 12 of gestation. During the treatment period, the animals were fed standard rodent food and water *ad libitum*. Pregnant rats were weighed on the 1^st^, 6^th^, 12^th^, and 20^th^ day of pregnancy (for day 20 experiment) and on the 1^st^, 6^th^, and 12^th^ day of gestation (for day 12 experiment).

### 2.2. Plant Material

Fresh leaves of khat (about 7000 g) were purchased at a local market in Aweday, 515 km east of Addis Ababa, Ethiopia, where it is commonly grown. The fresh bundles were packed in plastic bags and transported in an icebox to the laboratory. The fresh leaves were immediately kept in a deep freezer (-20°C). Plant identification, by a taxonomist, was carried out at the National Herbarium, College of Natural and Computational Sciences, Addis Ababa University, Ethiopia, and a voucher specimen (collection number SB-001) was deposited for future reference.

### 2.3. Preparation of Crude Extract

The leaves were chopped with a knife in a dark place, weighed by electronic digital balance, and put into an Erlenmeyer flask containing 80% methanol. Enough volume of solvent was added so as it covers the crushed plant material in the flask; then, the flask was sealed with aluminum foil and frequently agitated using a rotary shaker at 120 rpm for 24 h. It was then filtered using a Whatman No. 1 filter paper (90 mm diameter, Whatman Ltd, England) and macerated 3x for a total of 72 h and filtered. The combined filtrates were concentrated using a rotary evaporator (BUCHI Rotavapor R-205, Switzerland) at a temperature not exceeding 40°C. The concentrated extract was then transferred into a freeze-drying flask and lyophilized (OPERAN Lyophilizer, Korea). The dried extract was kept in a tightly sealed container at -20°C until use.

### 2.4. Experimental Animals

Nulliparous female albino Wistar rats weighing 200 to 230 g and sexually matured males of proven fertility were used. The rats were obtained from the animal breeding house of the Ethiopian Public Health Institute (EPHI). The rats were accommodated in stainless-steel cages where the temperature (22-23°C) and relative humidity (50% ± 10) of the room were controlled and 12 h of light and 12 h of dark were maintained. During the period of adaptation, all the animals received food (Wayne F6 Rodent Blox Pellets) and tap water. The handling of animals and all experimental procedures were conducted in compliance with the internationally accepted Organization for Economic Co-operation and Development (OECD) guideline [19], and the protocol was approved by the Institutional Review Board of EPHI. Based on the literature and previous studies, the animal species and model used in this study were believed to address the scientific objectives of the investigation. After 5 days of adaptation period, the animals were mate overnight by placing a male albino Wistar rat into a cage containing two nulliparous female rats. The male rat was introduced into the cage at about 17:00 hours. After an overnight mating, female rats were inspected for the presence of a copulatory plug the following morning and vaginal smears were taken for microscopic examination. If sperm cells are detected in the vaginal smear, it was considered as day 1 of gestation.

### 2.5. Grouping and Dosing of Animals

In both day 12 and day 20 experiments, a total of 50 pregnant rats were randomly divided into five groups. In each experiment, five rats were assigned to each group. Group I received vehicle and served as the pair-fed control group (CON). Groups II, III, and IV were treated with three doses of the crude khat extract: 250 mg/kg (K250), 500 mg/kg (K500), and 750 mg/kg (K750), respectively. Group V served as the untouched *ad libitum* control group and received the pellet diet and water unrestricted. Each animal in the experimental groups (Groups II, III, and IV) was given the respective doses of khat and the pellet diet and water *ad libitum*. The pair-fed control group animals were restricted-fed and received an equal amount of the same pellet diet and water only. The treatment period was from day 6 to day 12 of gestation, since this period is an active period of organ formation in rats [19].

The three treatment doses of khat extract were chosen based on previous study reports. The khat extract was weighed and mixed with 1.0 ml of the vehicle, and gavage was used for oral administration. Such route of administration was selected since the khat leaves are usually consumed orally. In addition, pharmacokinetic studies have shown that khat is readily absorbed into the blood from the gastrointestinal system [10].

### 2.6. Day 12 Khat Experiment

This experiment was planned to investigate possible embryotoxic effects of khat on rat embryos. On day 12 of gestation, at 12:00 hours, the animals were anesthetized with an intraperitoneal injection of pentobarbital. The gravid uterine horns were taken out of the abdomen and placed in a Petri dish containing normal saline. Each horn of the uterus was incised along the antimesometrial border to disclose the embryos covered with membranes. The surrounding embryonic membranes were curiously removed by using fine forceps. Thereupon, the visceral yolk sac was revealed and yolk sac circulation was examined. Once the embryonic membranes were removed, the embryos were detached from the corresponding placenta. Then, different body systems of the embryos were evaluated for developmental delays, namely, circulatory, nervous, visual, auditory, olfactory, and skeletal systems as well as the craniofacial region. All embryos were evaluated for developmental delays according to the Brown and Fabro morphological scoring system [20], which was adopted for use in *in vivo* investigation [16]. In addition, the numbers of somites were also counted.

### 2.7. Day 20 Khat Experiment

This experiment was designed to study gross congenital anomalies (fetotoxic effects) caused by exposure to khat in pregnant rats. On gestational day 20, gravid females were anesthetized with an intraperitoneal injection of pentobarbital; the uterine horns were exposed and examined intact. The number of implantation sites (yellowish nodules found on the mesometrial margin of uterine horns) and resorptions (metrial nodules not occupied by living or recently dead fetuses) was counted. Live or dead fetuses were determined by applying a gentle pressure on them. The fetuses, covered with membranes and attached with placenta, were disclosed by incising the uterine horns along the antimesometrial border. Then, each fetus was detached from the corresponding placenta, and its weight as well as crown-rump length (CRL) was measured. The weight of the placenta also was recorded. Following these measurements, all fetuses were examined for gross external malformations.

#### 2.7.1. External Evaluation

Gross external examination was conducted to check for possible treatment-related external malformations in the limbs, vertebral column, tail, external genitalia, and craniofacial regions of the fetuses.

#### 2.7.2. Histopathological Studies of the Placenta

Three to four placentae from each group were randomly selected and put in 10% formalin. The fixed placentae were transferred into 70% ethyl alcohol overnight. Following routine processing for light microscopy, the placentae were blocked in paraffin and cut into sections of 4 *μ*m thickness using a Sorvall-JB microtome. The sections were then placed on glass slides, dewaxed, dehydrated, and stained with hematoxylin and eosin (H&E). Finally, the stained slides were covered with a coverslip for microscopic examination. The sections were examined for evidence of structural and vascular alterations using a Nikon binocular light microscope. Decidual zone, labyrinthine zone, giant cells, and trophoblasts of the placenta were examined and used as indices of functional as well as structural changes in the placenta.

#### 2.7.3. Light Microscopy and Photography

Stained tissue sections of the placenta were carefully examined by a pathologist under a binocular compound light microscope. Tissue sections from the experimental groups were examined for any evidence of histopathological changes with respect to those of the controls. After examination, representative photomicrographs were taken by using an automated built-in digital photo camera (EVOS XL, USA) under a magnification of ×20 objective.

### 2.8. Data Analysis

The data, concerning weight gain, pregnancy outcomes, maternal food intake, fetal growth, and developmental anomalies in each group, were analyzed by one-way analysis of variance (ANOVA) using the SPSS version 20. The data regarding embryonic development and histopathological analysis was analyzed by using chi-square analysis.

## 3. Results

### 3.1. Day 12 Experiment

Treatment of pregnant rats with khat at doses of 250 mg/kg, 500 mg/kg, and 750 mg/kg body weight, from days 6 to 12 of gestation produced a dose-dependent decrease in body weight gain of the dams. The decrease in the body weight gain was significant in all khat-treated groups compared to that in the control group (Table 1).

Maternal daily food intake was also significantly reduced during the treatment period in all treatment groups and the pair-fed control compared to that in the *ad libitum* control group. The K750 showed a significantly higher incidence of fetal resorptions and also a significant decrease in the number of implantation sites compared to the pair-fed control and *ad libitum* groups (*p* < 0.05) (Table 1).

#### 3.1.1. Embryonic Development

**Figure 1:**
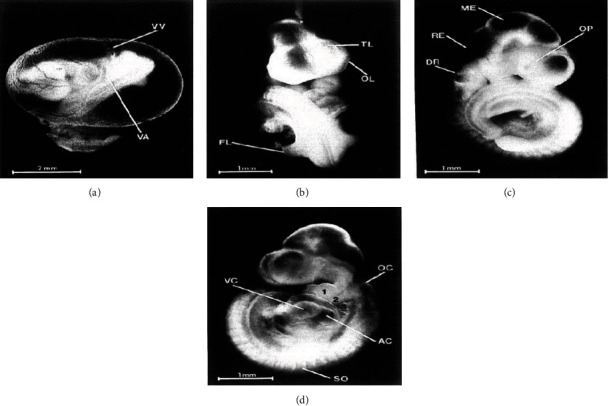
Twelve-day-old rat embryos showing various organ primordial: Brown and Fabro [20]. (a) An embryo enclosed in the yolk sac, and yolk sac circulation showing vitelline artery (VA) and vitelline vein (W); (b) with the yolk sac removed, the embryo shows telencephalon (TL), olfactory plate (OL), and forelimb (FL); (c) an embryo showing optic primordia (OP), mesencephalon (ME), rhombencephalon (RE), and dorsal recess of otocyst (DR); and (d) an embryo showing otocyst (OC), atrium commune (AC), ventriculus communis (VC), and somite (SO).

The developmental status of the primordia of the different systems was evaluated based on the morphological scoring system developed by Brown and Fabro. The degree of yolk sac circulation and heart development was significantly affected (*p* < 0.05) in embryos of K500 and K750 groups (Table 2 and Figure 1).

The development of forebrain, midbrain, hindbrain, and caudal neural tube was not affected across all treatment groups. Embryonic development of the otic system, optic system, olfactory system, and brachial bars was significantly delayed in treatment groups of K500 and K750 compared to the pair fed control and *ad libitum* groups (*p* < 0.05) (Tables 2 and 3; Figure 1).

The development of maxillary process, forelimb, and hindlimb was significantly affected in the treatment group of K750 (*p* < 0.05) compared to the pair-fed control and *ad libitum* groups. However, the development of mandibular process was not affected (Tables 3 and 4, Figure 1). The number of somites, the indices of musculoskeletal system development, was affected in the K750 group compared to that in the control groups but was not statistically significant (Table 4).

### 3.2. Day 20 Experiment

Maternal weight gain in all the treatment groups was significantly lower (*p* < 0.05), and the number of fetuses also was significantly decreased (*p* < 0.05) in the K750 mg/kg group compared to the pair-fed control and *ad libitum* groups. Implantation sites and the number of live fetuses per litter were significantly reduced at the dose of 750 mg/kg compared to that in both the pair-fed control and the unrestricted-fed *ad libitum* groups (*p* < 0.05) (Table 5). The number of fetal resorptions and dead fetuses per litter was higher at the dose of khat 750 mg/kg compared to that in the pair-fed control and *ad libitum* groups, but the relationship was not statistically significant.

**Figure 2:**
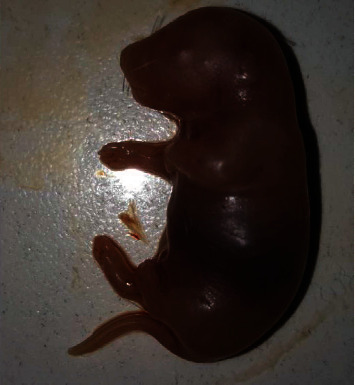
Near-term rat fetus treated with khat.

The growth of 20-day-old fetuses was significantly altered by treatment with a high dose (K750) of khat extract compared with pair-fed and *ad libitum* control groups. Both CRL and fetal weight showed a significant decrease in the high-dose treated group (K750) (Table 6). No gross external developmental abnormalities were observed in any of the regions examined (Figure 2).

#### 3.2.1. Histopathological Analysis of the Placenta

**Figure 3:**
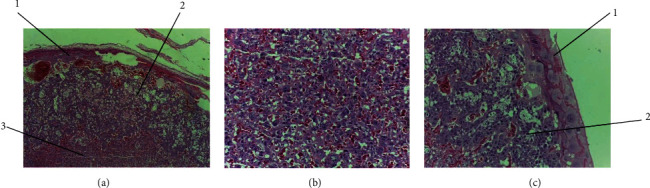
Photomicrographs of the placenta of control group rats showing normal structural architecture: (a) decidual (1), trophoblastic (2), and labyrinth zone (3); (b) labyrinth zone of rat placenta; (c) decidual (1) and trophoblastic zone (2) of rat placenta; H&E stain, ×100.

There was no significant variation in either weight change or gross anomalies of the placenta observed between khat-treated and control groups. However, light microscopic examination of the placentae from treatment groups showed structural changes and differences compared to the *ad libitum* and pair-fed control groups. Dose-dependent pathological changes in the placenta were observed. These microscopic structural changes were seen in the decidua basalis, trophoblastic zone, and labyrinth zone (Figure 3).

**Figure 4:**
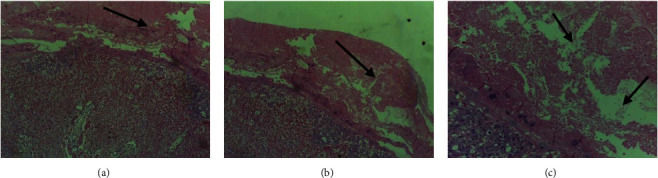
Photomicrographs of the placenta of khat-treated rats (500 mg/kg) showing decidual hypertrophy (a, b) (arrow) and necrosis (f) (arrow); H&E stain, ×100.

**Figure 5:**
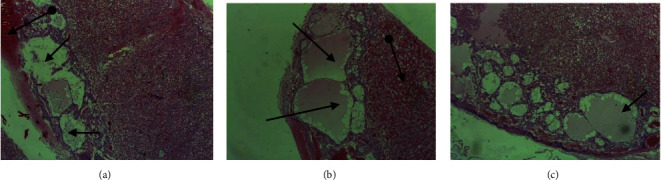
Photomicrographs of the placenta of khat-treated rats (750 mg/kg) showing cystic changes on the decidua (arrow) and hemorrhage on the decidua and labyrinth zones (headed arrow) (a–c); H&E stain, ×100.

Sections obtained from treatment groups revealed multiple lesions that included decidual hypoplasia and necrosis, cytolysis, apoptosis, and vascular degeneration (Table 7). In the labyrinth, the trophoblasts showed necrosis and disintegration of the placental membrane that separate maternal blood from embryonic capillaries, resulting in admixing of maternal and fetal blood (Figure 4). Furthermore, vascular congestion and extensive areas of hemorrhage were observed in the labyrinth (Figure 5).

## 4. Discussion

The findings of the present study showed that exposure to hydroalcoholic khat extract causes embryotoxic/fetotoxic effects in rats, and the effects were dose-related.

Embryonic development was assessed according to the morphological scoring system of Brown and Fabro [20]. A statistically significant delay in the development of the embryo at doses 500 mg/kg and 750 mg/kg of khat was observed. Delayed development of the heart, otic system, optic system, and olfactory system was seen. Development of primordia of forelimb, hindlimb, and brachial bars was also significantly delayed in the khat-treated groups. The development of the musculoskeletal system, as indicated by the number of somites, was also affected in the khat-treated groups but yet not statistically significant. The development of the central nervous system including caudal neural tube, forebrain, midbrain, and hindbrain development and development of maxillary process were not significantly affected at all doses of khat treatment.

Pregnancy outcomes also were affected in khat-treated pregnant rats. Maternal weight gain was lower in animals treated with K500 and K750 compared to the *ad libitum* group. This finding is consistent with other animal and human studies which reported that khat exposure during pregnancy resulted in decreased maternal weight gain [12, 21]. Earlier studies tried to relate the decreased maternal weight gain with the anorexic effects of khat [21]. In the present study, however, the reduced weight gain was observed throughout the gestational period, even though the treatment with khat extract was only up to the 12^th^ day of gestation.

Prenatal growth retardation was observed in near-term fetuses of khat-treated rats in excess of those in the pair-fed control group and *ad libitum* group. Significant reductions in fetal weight and crown-rump length were observed at all doses of khat treatment. This observation is in line with few human and animal studies which reported significant reduction in birth weight of newborns of khat users, respectively [22, 23].

A significant decrease in the number of fetuses and implantation sites was observed when animals were treated with the highest dose of 750 mg/kg.

In the present study, developmental anomalies and gross external organ malformations were not observed in all animals at all doses of khat treatment.

During implantation of the embryo, invasion of the uterine wall by syncytiotrophoblast cells of the placenta is critical and finely controlled. Insufficient invasion of maternal vasculatures has been associated with placental pathologies such as fetal growth restriction, whereas an excessive trophoblast invasion is correlated with choriocarcinoma [24]. The placenta is a hugely vulnerable target organ for chemical-induced toxic insults, and various placenta toxic agents have been reported. Histopathological investigations of the placenta play a central role in the understanding of the mechanism of embryotoxicity and developmental toxicity and could benefit reproductive toxicity studies [25, 26].

According to the current study, exposure to khat during pregnancy caused disorganization of the vascular tree in the labyrinth layer (the major site of nutrient and gas exchange), with less branching in the treatment group at the highest dose (Figures 4(a) and 5(a)). The observed disorganization of the vascular tree in the labyrinth layer could explain the fetal growth retardation seen in khat-treated rat fetuses and the intrauterine fetal death (IUFD), since this region of the placenta is the major site where nutrient and gas exchange occurs.

Decidua basalis, thin layer at the base of the placenta, is an important site for maternal angiogenesis. The decidua basalis includes newly developed blood vessels, which play essential roles in the development of vascularized decidual-placental interface. The decidua can produce a wide range of hormones, cytokines, growth factors, and immunomodulatory molecules involved in the recruitment of the limited but specific immune cell populations and growth of the placenta [26]. Findings of the current study showed moderate to severe decidual hypertrophy, atrophy, necrosis and cystic formation, and cytolysis (Figures 4 and 5). These findings may be explained by the possible causes of placental hypertrophy, including drug- and chemical-induced compensatory placental hypertrophy, a compensatory reaction to intrauterine growth retardation (IUGR) under the unfriendly maternal environment, diminished capacity of uteroplacental circulation to transfer nutrients to the fetus.

## 5. Conclusion

In the present investigation, the embryotoxic/fetotoxic and toxic effects of khat were evidenced by a significant delay in embryonic and fetal development and decreased maternal weight gain during the period of gestation. This study, for the first time, has also demonstrated that khat has pathological effects on histological features of rat placenta at a cellular level. Based on the presented data, it can be concluded that administration of crude extract of khat to pregnant rats may not be safe. Since embryotoxic/fetotoxic effects of khat have now been established experimentally, its use, especially during pregnancy, should be strongly discouraged.

## Figures and Tables

**Table 1:** Pregnancy outcomes of day 12 khat experiment.

Treatment group	Maternal weight gain per litter (g)	Implantation sites per litter	Resorptions per litter
Group I (control) *n* = 5	14.2 ± 0.01	9.8 ± 0.8	0.2 ± 0.4
Group II (K250) *n* = 5	4.9 ± 0.4^a^	9.8 ± 0.8	0
Group III (K500) *n* = 5	3.9 ± 4.9^a^	9.6 ± 1.1	0.2 ± 0.4
Group IV (K750) *n* = 5	3.1 ± 4.5^a^	7.8 ± 0.8^b^	0.6 ± 0.9^a^
Group V (*ad libitum* control) *n* = 5	14.6 ± 2.9	10.2 ± 0.8	0

K250: khat extract 250 mg/kg; K500: khat extract 500 mg/kg; K750: khat extract 250 mg/kg; *n*: number of pregnant rats; results are summarized as mean ± SDM; ^a^significantly different (*p* < 0.05) from the *ad libitum* group and pair-fed control group at all doses of treatment; ^b^significantly different (*p* < 0.05) from the *ad libitum* control group; one-way ANOVA.

**Table 2:** Embryonic circulatory and auditory system development following treatment of pregnant rats with khat: day 12 experiment.

Treatment group	Percent retarded development		
	Yolk sac	Heart	Otic system
Control	0	0	2.6
K250	0	1.7	2.6
K500	8.5^a,b^	3.4	7.8^a,b^
K750	5.6^a,b^	10^a,b^	12.2^a,b^
*Ad libitum* control	0	0	2.2

K250: khat extract 250 mg/kg; K500: khat extract 500 mg/kg; K750: khat extract 250 mg/kg; results are summarized as percentages of retarded development; ^a,b^significantly different (*p* < 0.05) from *ad libitum* control and pair-fed control groups; chi-square.

**Table 3:** Embryonic craniofacial development after treatment of pregnant rats with khat: day 12 experiment.

Treatment group	Percent retarded development			
	Optic system	Olfactory system	Branchial bars	Maxillary process
Control	0	1.7	4	4
K250	0	3	5	6
K500	5.6^a, b^	4.1	7	8
K750	8.2^a,b^	10^a,b^	10^a,b^	10^a,b^
*Ad libitum* control	1.3	1.5	4	4

K250: khat extract 250 mg/kg; K500: khat extract 500 mg/kg; K750: khat extract 250 mg/kg; results are summarized as percentages of retarded development; ^a,b^significantly different (*p* < 0.05) from *ad libitum* control and pair-fed control groups; chi-square.

**Table 4:** Embryonic musculoskeletal system development following treatment of pregnant rats with khat: day 12 experiment.

Treatment group	Somite number	Forelimb	Hindlimb
Control	39.2 ± 0.9	0	0
K250	38.9 ± 0.8	0	1
K500	37.8 ± 1.1	0	1
K750	35.8 ± 1	8.3^a^	32^a^
*Ad libitum* control	39.2 ± 1.9	0	0

K250: khat extract 250 mg/kg; K500: khat extract 500 mg/kg; K750: khat extract 250 mg/kg; results are summarized as mean ± SDM (ANOVA) or percentages of retarded development (chi-square); ^a^significantly different (*p* < 0.05) from *ad libitum* control and pair-fed control groups.

**Table 5:** Pregnancy outcomes of day 20 khat experiment.

Pregnancy outcomes		Group (*n* = 5/group)				
		Group I (control)	Group II (K250)	Group III (K500)	Group IV (K750)	Group V (*Ad libitum* control)
Maternal weight gain	Day 6-12	16.3 ± 0.3	12.4 ± 1.3^a^	8.8 ± 0.1^a^	9.9 ± 2.1^a^	16.5 ± −2.3
	Day 13-20	63.8 ± 1.5	55.3 ± 1.3	42.2 ± 11.4^b^	41.3 ± 1.6^b^	61.0 ± −3.3
No. of fetuses		49.0	49.0	46.0	33.0^b^	49.0
Implantation sites/dam		10.0 ± 0.7	10.0 ± 1.0	9.8 ± 0.4	7.8 ± 0.4^b^	10.0 ± 1.2
Number of live fetus/litter		9.8 ± 0.8	9.6 ± 1.1	9.2 ± 0.8	6.2 ± 0.8^b^	9.8 ± 1.1
Number of resorptions/litter		0.2 ± 0.4	0.4 ± 0.5	0.6 ± 0.8	1.2 ± 1.3	0.2 ± 0.4
Number of dead fetus/litter		0	0	0	0.4 ± 0.5	0

Results are summarized as mean ± SDM; ^a^significantly different (*p* < 0.01) from *ad libitum* and pair-fed control groups, at all doses of khat; ^b^significantly different (*p* < 0.05) from *ad libitum* and pair-fed control groups, one-way ANOVA. *n*: number of pregnant rats.

**Table 6:** Growth of rat fetus following treatment with khat.

Treatment group	Fetal weight (g)	Crown-rump length (cm)
Group I, control pair-fed	4.9 ± 0.2	3.6 ± 0.2
Group II, K250	4.8 ± 0.3	3.7 ± 0.2
Group III, K500	4.7 ± 0.3	3.5 ± 0.1
Group IV, K750	3.9 ± 0.3^a^	3 ± 0.2^a^
Group V, *ad libitum* control	5 ± 0.1	3.8 ± 0.2

K250: khat extract 250 mg/kg; K500: khat extract 500 mg/kg; K750: khat extract 250 mg/kg; results are summarized as mean ± SDM; ^a^significantly different (*p* < 0.01) from *ad libitum* and pair-fed control groups; one-way ANOVA.

**Table 7:** Comparison of histopathological alterations in rat placenta.

Group	Percent of placental abnormalities				
	Decidual necrosis	Cytolysis	Necrosis	Apoptosis	Severe decidual hypoplasia/atrophy
Control	0	0	0	0	0
K250	25	25	0	0	0
K500	50	100^a^	0	25	0
K750	50	50	25	50	25
*Ad libitum* control	0	0	0	0	0

K250: khat extract 250 mg/kg; K500: khat extract 500 mg/kg; K750: khat extract 250 mg/kg; results are summarized as percentages of placental abnormalities; ^a^significant difference seen from the *ad libitum* and pair-fed control groups (*p* < 0.05), chi-square.

## Data Availability

All data are included in the manuscript.

## Abbreviations

EPHI: Ethiopian Public Health Institute
H&E: Hematoxylin and eosin
IRB: Institutional Review Board
OECD: Organization for Economic Co-operation and Development
SPSS: Statistical Package for Social Science
ANOVA: Analysis of variance
CON: Control group
K250: Khat 250 mg/kg
K500: Khat 500 mg/kg
K750: Khat 750 mg/kg
CRL: Crown-rump length
IUFD: Intrauterine fetal death
IUGR: Intrauterine growth retardation.

## References

[1] Gebissa E. (2010). Khat in the Horn of Africa: historical perspectives and current trends. *Journal of ethnopharmacology*.

[2] Selassie S. G., Gebre A. (1996). Rapid assessment of drug abuse in Ethiopia. *Bulletin on Narcotics*.

[3] Pantelis C., Hindler C. G., Taylor J. C. (1989). Use and abuse of khat (*Catha edulis*): a review of the distribution, pharmacology, side effects and a description of psychosis attributed to khat chewing. *Psychological medicine*.

[4] Hassan N. A., Gunaid A. A., El-Khally F. M., Murray-Lyon I. M. (2002). The effect of chewing khat leaves on human mood. *Saudi Medical Journal*.

[5] Alele P. E., Ajayi A. M., Imanirampa L. (2013). Chronic khat (*Catha edulis*) and alcohol marginally alter complete blood counts, clinical chemistry, and testosterone in male rats. *Journal of experimental pharmacology*.

[6] Kennedy J. G., Teague J., Rokaw W., Cooney E. (1983). A medical evaluation of the use of qat in North Yemen. *Social Science & Medicine*.

[7] Nigussie T., Gobena T., Mossie A. (2013). Association between khat chewing and gastrointestinal disorders: a cross sectional study. *Ethiopian journal of health sciences*.

[8] Alsalahi A., Abdulla M. A., al-Mamary M. (2012). Toxicological features of *Catha edulis* (khat) on livers and kidneys of male and female Sprague-Dawley rats: a subchronic study. *Evidence-Based Complementary and Alternative Medicine*.

[9] Dhaifalah I., Santavy J. (2004). Khat habit and its health effect. A natural amphetamine. *Biomedical papers of the Medical Faculty of the University Palacky, Olomouc Czech Republic*.

[10] Wabe N. T. (2011). Chemistry, pharmacology, and toxicology of khat (catha edulis forsk): a review. *Addict Health.*.

[11] Kedir H., Berhane Y., Worku A. (2013). Khat chewing and restrictive dietary behaviors are associated with anemia among pregnant women in high prevalence rural communities in eastern Ethiopia. *PloS one.*.

[12] ACMD (2005). *Khat (Qat): Assessment of Risk to the Individual and Communities in the UK, advisory council on the misuse of drugs home office*.

[13] Hoffman R., Al’Absi M. (2010). Khat use and neurobehavioral functions: suggestions for future studies. *Journal of ethnopharmacology*.

[14] Kuczkowski K. (2004). Re: cathinone: a new differential in the diagnosis of pregnancy induced hypertension. *East African medical journal*.

[15] Eriksson M., Ghani N., Kristiansson B. (1991). Khat-chewing during pregnancy-effect upon the off-spring and some characteristics of the chewers. *East African medical journal*.

[16] Seyoum G., Persaud T. (1995). Protective influence of zinc against the deleterious effects of ethanol in postimplantation rat embryos *in vivo*. *Experimental and Toxicologic Pathology*.

[17] Seyoum G. (2016). Influence of methionine supplementation on nicotine teratogenicity in the rat. *Ethiopian Pharmaceutical Journal*.

[18] Teshome D., Tiruneh C., Berihun G. (2021). Toxicity of methanolic extracts of seeds of *Moringa stenopetala*, *Moringaceae* in rat embryos and fetuses. *BioMed Research International*.

[19] OECD (2001). Guideline for Testing Chemicals: Prenatal Developmental Toxicity Study.

[20] Brown N. A., Fabro S. (1981). Quantitation of rat embryonic development in vitro: a morphological scoring system. *Teratology*.

[21] Abdel-Aleem M. A. (2015). Khat chewing during pregnancy: an insight on an ancient problem impact of chewing khat on maternal and fetal outcome among Yemeni pregnant women. *Journal of Gynecology and Neonatal Biology*.

[22] Abdul Ghani N., Eriksson M., Kristiansson B., Qirbi A. (1987). The influence of khat-chewing on birth-weight in full-term infants. *Social Science & Medicine*.

[23] Jansson T., Kristiansson B., Qirbi A. (1988). Effect of khat on maternal food intake, maternal weight gain and fetal growth in the late-pregnant guinea pig. *Journal of ethnopharmacology*.

[24] Holloway A. C., Salomon A., Soares M. J. (2014). Characterization of the adverse effects of nicotine on placental development: in vivo and in vitro studies. *American Journal of Physiology-Endocrinology and Metabolism*.

[25] Erdemli Z., Erdemli M. (2020). Vitamin E plays a protective role while acrylamide administration disrupted the placenta structure in pregnancy: an experimental study. *Annals of Medical Research*.

[26] Furukawa S., Hayashi S., Usuda K., Abe M., Hagio S., Ogawa I. (2011). Toxicological pathology in the rat placenta. *Journal of toxicologic pathology*.

